# Health-industry linkages for local health: reframing policies for African health system strengthening

**DOI:** 10.1093/heapol/czy022

**Published:** 2018-03-19

**Authors:** Maureen Mackintosh, Julius Mugwagwa, Geoffrey Banda, Paula Tibandebage, Jires Tunguhole, Samuel Wangwe, Mercy Karimi Njeru

**Affiliations:** 1 Faculty of Arts and Social Sciences, The Open University, Walton Hall, Milton Keynes MK7 6AA, UK; 2 Department of Science, Technology, Engineering and Public Policy, University College London, London W1T 6EY, UK; 3 Science, Technology and Innovation Studies, University of Edinburgh, Edinburgh EH1 1LZ, UK; 4 Economic and Social Research Foundation, Dar es Salaam, Tanzania; 5 Centre for Public Health Research, Kenya Medical Research Institute, Nairobi, Kenya

**Keywords:** Local production of medicines, Africa, access to medicines, health system, health-industry linkages, local health, global health

## Abstract

The benefits of local production of pharmaceuticals in Africa for local access to medicines and to effective treatment remain contested. There is scepticism among health systems experts internationally that production of pharmaceuticals in sub-Saharan Africa (SSA) can provide competitive prices, quality and reliability of supply. Meanwhile low-income African populations continue to suffer poor access to a broad range of medicines, despite major international funding efforts. A current wave of pharmaceutical industry investment in SSA is associated with active African government promotion of pharmaceuticals as a key sector in industrialization strategies. We present evidence from interviews in 2013–15 and 2017 in East Africa that health system actors perceive these investments in local production as an opportunity to improve access to medicines and supplies. We then identify key policies that can ensure that local health systems benefit from the investments. We argue for a ‘local health’ policy perspective, framed by concepts of proximity and positionality, which works with local priorities and distinct policy time scales and identifies scope for incentive alignment to generate mutually beneficial health–industry linkages and strengthening of both sectors. We argue that this local health perspective represents a distinctive shift in policy framing: it is not necessarily in conflict with ‘global health’ frameworks but poses a challenge to some of its underlying assumptions.

## Introduction

In international research and policy debates, health system strengthening and industrial development have been generally addressed within two separate silos (Mackintosh *et al.* 2007, 2016). When health–industry linkage in sub-Saharan Africa (SSA) has been raised, each silo has viewed the proposition with scepticism. International health experts have doubted Africa-based manufacturers’ capabilities to supply competitively priced, good quality medicines on a timely basis (Kaplan and Laing 2005; Seiter 2005; Kaplan *et al.* 2011; Wilson *et al.* 2012), fearing therefore a negative impact on medicines access. Industrialization experts meanwhile have focused on promotion of lower technology export sectors and primary product processing in Africa, by implication regarding pharmaceuticals as too complex with too high a learning curve and regulatory/governance requirements (Dinh *et al.* 2012; Lin 2013). Public health research meanwhile has largely ignored industrialization as a social determinant of health (CSDH 2008; Battams and Matlin 2013), while research on health systems strengthening has lumped industry into a general category of other relevant input sectors (Bigdeli *et al.* 2014).

Key Messages

- The health system benefits of local production of pharmaceuticals in SSA are contested, while access to medicines remains generally poor.
- A current wave of pharmaceutical industry investment offers an opportunity to link industrialization to improved access to medicines.
- A ‘local health’ policy perspective can identify policies for health–industry linkages that benefit both health systems and industrial development.

However policy makers, industrialists and researchers in Africa are increasingly exploring and promoting synergies between local industrial production of pharmaceuticals and medical supplies and improvement in coverage and quality of health care, especially for low-income populations (Government of Uganda 2002; Republic of Ghana 2004; African Union 2007, 2012; Berger *et al.* 2010; Government of Kenya 2010; EAC 2011; FDRE 2015; Gebre-Mariam *et al.* 2016; URT 2016). International organizations have responded: the World Health Organization (WHO) strategic framework for medicines and health products (WHO 2017a: 8, 12) recognizes the relevance of local manufacturing of quality medicines and health products for access, a view reflecting collaborative UN research and policy (UNCTAD 2011; WHO 2011a; Sidibé *et al.* 2014). Linkages between health policy and industrial change in low and middle income country (LMIC) contexts more broadly are increasingly researched (Srinivas 2012; Shadlen and Massard da Fonseca 2013).

Meanwhile, low-income populations in SSA continue to suffer severely inadequate and exclusionary health care undermined by poor access to medicines and supplies (Wagner *et al.* 2011; WHO 2011b; Bigdeli *et al.* 2014; Wirtz *et al.* 2017). Median availability of essential medicines 2007–14 was only 60% overall, and 56% in the public sector of low/lower-middle-income countries (WHO 2017b: 11), and has changed little in SSA countries (UN 2015: 55–6), despite major funding efforts for HIV and TB medication (IHME 2017). Better access and more appropriate use are required for all the aspects of health system strengthening in the Universal Health Coverage (UHC) 2030 Joint Vision (WHO/World Bank 2017): for reducing severe inequity, and improving quality, responsiveness, efficiency and resilience.

This article draws on recent fieldwork to address the implications for global health of the shift in African perspectives on health–industry linkages, a shift embedded in a wider policy narrative on building resilient, inclusive and sustainable economic systems (African Union 2014a). We first outline a ‘local health’ framework emerging from interviews and data collection, mainly in Tanzania and Kenya. We then trace how this perspective embeds health system strengthening within local industrial–health system linkages and wider economic, technological and industrial development. Finally, we discuss the implications for global health perspectives.

## ‘Local health’: reframing health system strengthening

A concept of ‘local health’, as it emerges in our interviews and current African policy debate, is rooted in a dialogue between *proximity* and *positionality*. ‘Proximity’ refers to cumulative local interactions and mutual influences arising from co-location (Boschma 2005). ‘Positionality’ (Rowson *et al.* 2012) refers to the influence of location of agency on the framing of issues and priorities, with attendant claims to power and legitimacy in policy making.

Proximity can usefully be analysed on three dimensions: geographical proximity, relational proximity and the values assigned to proximity (Eriksen 2013). In health research, geographical proximity is measured as a determinant or index of accessibility of services; in industrial development, as an explanatory factor of industrial clustering of related industries. In health–industry linkages, geographical proximity potentially generates more rapid supply response. Relational proximity echoes the health literature‘s recognition of local culture as an important determinant and contributor to health services’ response to population needs (Hahn and Inhorn 2009); in industry, it reflects what has been called industrial ‘atmosphere’, the cumulative benefits of local learning and spillovers of tacit knowledge (Ravix 2014) and relationships with universities and government. In health–industry linkages, it reflects the scope for a more agile response to local needs within local economies. Finally, the values given to proximity can be picked up in mutual understanding, legitimation and trust in known health care providers; in collaboration between input and final product producers; and in merited trust in locally produced health care products.

Positionality, defined by local power, agency and responsibility, is reflected in locally distinctive priorities and in sharply differentiated views—as compared to global health approaches—on risk, security and timescales for policy making. For example, local interviews on health supplies emergency planning priorities focused on day-to-day immediate emergency needs, while for pandemic preparedness, a central concern was local scientific competence and production capacity, recognizing a positionality-derived imperative on governments to protect their own populations first.

These distinctive local concerns pull closer together policies for industry, science and health around strengthening security of pharmaceutical supplies for local health care. They interconnect risk management with local health security, safety and responsibility. As Giddens (1999: 7–9) argues, in contexts of uncertainty and innovation, risk and responsibility are closely interrelated. For local policy makers to assume greater responsibility for medium term risk management requires the building of greater technical and organizational capability in health- and industry-related skills. Increasingly this imperative is framed in terms of ownership: ‘To be able to generate wealth and give its future generations a chance, Africa must take ownership of its health’ (Lopes 2014). Positionality thus invokes claims of legitimacy for policy and practice.

We trace in our findings the implications of this ‘local health’ perspective in local health policy, and the emerging interconnections with industrial change. We document the locally perceived relevance of local production for health, and explore the scope for incentive alignment across sectors. The Findings subsections thus identify what potential health benefits from industrial proximity are locally recognized; note current policy scope for exploiting those synergies; and identify areas where incentivizing industrial development in pharmaceuticals and supplies can also incentivize responsiveness to health sector needs, and conversely where reshaping procurement can open markets for local firms, in an incentive–compatible spiral of improvement. 

## Methods

Research on health system–industry relationships in 2012–15^1^ included a first phase (2012–13) using a convergent parallel mixed methods research design (Ozawa and Pongpirul 2014). A total of 160 qualitative interviews were conducted in 4 districts in Tanzania, and 4 counties in Kenya, purposively selected to represent a wide range of geographical area, incomes, infrastructure and health outcomes, including 2 urban and 2 rural locations in each country. Health facilities, pharmacies and drug shops (42 in Tanzania and 55 in Kenya) were purposively selected from public (32), faith-based/NGO (17) and private (48) sectors. Semi-structured interviews covered procurement and supply processes and opinions on local vs imported supplies, for medicines, medical supplies and equipment, laboratory supplies and basics such as bed nets, sheets and cleaning materials, using open-ended questions that invited free expression and avoided leading interviewees. Quantitative data were also collected on availability and source of a set of tracer essential medicines, supplies and equipment on the day of the visit (listed in online Supplementary Data— Supplementary data are available at *Health Policy and Planning* online, and selected with advice from local regulators and pharmaceutical experts).

In a second phase (2013–14), locally based manufacturers of medicines, medical and other supplies, and of inputs such as packaging (11 in Tanzania and 12 in Kenya) were interviewed, and 29 interviews conducted with government officials, wholesalers, procurement agents, regulators, government officials and manufacturing associations. Topics included business histories and strategies, trading conditions, case studies of domestic market supply decisions, and policy, regulatory and procurement frameworks and experience.

In April–May 2017, 23 further interviews^2^ in Tanzania, Kenya and Uganda on local pharmaceutical production and its implications for health care included 6 manufacturers, 3 wholesalers (procurement agencies and private distributors), 5 regulatory bodies and government ministries; 7 clinicians, pharmacists and local pharmaceutical consultants and 2 East African Community (EAC) officials. We also met a group of senior informants at The Global Fund in Geneva. This article also draws on discussions with a broader network of African experts in the context of meetings and consultations, as well as secondary literature.

The quantitative data were analysed using Stata 11. The qualitative data were coded and sorted into key themes using NVivo software, then systematically analysed for distinct concepts and arguments. This article presents mainly qualitative findings, triangulated where appropriate with quantitative data. While the data set is original and substantial, the quantitative findings of the 2012–13 research are not statistically representative for either country.

Ethical clearance was obtained from the Open University Human Research Ethics Committee in the UK, the Kenyatta National Hospital Ethical Review Board in Kenya and the National Institute for Medical Research Ethics Review Committee in Tanzania. All participants consented to the research, having been assured that participation was voluntary and that their anonymity would be preserved in published research findings.

## Findings

### Is proximity in source of supplies a health policy concern?

In Tanzania and Kenya, health sector informants consistently argued that local manufacturing was relevant for availability of supplies, and could improve it further, citing short supply chains, distribution in rural areas, and scope for closer regulation and scrutiny than of overseas suppliers. In both countries over half of medicines are accessed by private payment, while local manufactures’ prices fluctuate with competitive market conditions and may be higher or lower than Indian import prices (Mackintosh and Mujinja 2010; ACT Watch *et al.* 2017; Ewen *et al.* 2017).

In both countries, some local firms had built brand recognition and trust from clinicians and the public, e.g.:

*Our patients … appreciate the products from Cosmos* (Faith-based health centre, Kenya).

*Shelys has good-quality drugs which are readily available and the price is affordable* (Public health centre, Tanzania).

These firms are long established, with wide distribution networks, and local production was seen as important for rural access. Availability of basic medication is consistently worse in rural areas, reflecting delivery difficulties and low demand due to very low incomes (Cohen *et al.* 2010; Mackintosh and Mujinja 2010; URT 2014). In Kenya, local products were widely preferred over Indian imports where European imports were unaffordable, e.g.:

*… the locally manufactured drugs are cheap …. mission hospitals, clinics, district hospitals and local pharmacists in upcountry, they really support local manufacturers* (Private hospital Kenya).

Data on availability supported these assertions: in both countries local products formed a higher share of tracer medicines available on rural than on urban shelves in 2013 (Table 1).

**Table 1.** Geographical source of tracer essential medicines available on day of visit, facilities and shops, all sectors, by rural/urban, Tanzania and Kenya, 2013 (% of total by rural/urban location)

Manufacturing location	Tanzania		Kenya	
	Local	External	Local	External
Rural	19.8	80.2	54.9	45.1
Urban^a^	13.0	87.0	35.5	64.5

*Source:* Calculated from fieldwork data 2013. Tanzania *n* = 646; Kenya *n* = 1043.

a In Tanzania, includes semi-urban areas on outskirts of cities and small urban areas in rural districts.

In Tanzania, locally manufactured essential medicines have been shown to reach rural locations more effectively than imported items (Mujinja *et al.* 2014). Local manufacturers stated that they relied on active rural distribution, using their own logistics, and one firm in 2017 was actively expanding its in-house distribution capability and brand advertising to widen its market. Closing a rural/urban availability gap for subsidized imported antimalarial combination therapy (ACTs) had required active interventions in distribution, including rural subsidies in Kenya (Cohen *et al.* 2010; Morris *et al.* 2015; ACT Watch *et al.* 2017).

In both countries health sector informants in 2012–13 complained about two aspects of local product quality: hardness—a tendency for tablets to disintegrate before use—and poor packaging quality. Local manufacturers also identified a problem with low quality of local packaging suppliers.

Finally, when asked in 2017 about supplies for emergency preparedness, all health sector respondents in Kenya and Tanzania advocated local production of medicines to address day-to-day lethal emergencies, mentioning saline drips for cholera, oral rehydration salts (ORS) for childhood diarrhoea, oxytocin for maternal haemorrhage, hydrocortisone, magnesium sulphate and adrenaline for allergic reactions, asthma and pre-eclampsia. Shortages of these items repeatedly cause emergency deaths. When asked about pandemic preparedness, African stakeholders interviewed in 2017 took a medium-term view, specifying building local scientific, technological and production capabilities, including local vaccine manufacturing, intellectual property (IP)-linked partnerships with multinationals, and use of flexibilities under the Agreement on Trade-Related Aspects of Intellectual Property Rights (TRIPS).

### Should local health policy consider industrial benefits?

Some health sector interviewees in 2013 recognized broader social and economic benefits from enhanced local production:

*There are a lot of benefits, for example …. you’ll create employment by virtue of them manufacturing in this country* (Private pharmacy, Kenya).

*Local pharmaceutical industries have been stimulated and so have created employment* (Drug shop, Tanzania).

Growing appreciation of these wider benefits was evident among health sector interviewees in 2017 in Tanzania. Like many African countries, Tanzania has shifted national policy emphasis towards industrialization, including pharmaceutical manufacturing as a priority sector in its Five-Year Development Plan 2016–2021, aiming explicitly to improve health care and enhance access to medicines (URT 2016: 49). A Tanzanian Health Ministry official said in 2017: ‘we used to think very narrowly’, just health needs, but now they also consider industrial benefits to Tanzania.

Health policy makers interviewed were thus aware of scope to encourage, influence and take advantage of a current wave of pharmaceutical investment, to respond to local health needs.

Across the East African region, the scope for health policy linkages with industry is expanding with new foreign and local investment. Tanzanian Health Ministry officials in 2017 were fielding enquiries from potential overseas investors, including UAE, Egypt, India, Pakistan and China. In Tanzania and Kenya, the most prominent firm has recently been subject to overseas takeover: in Tanzania, Aspen, a South African multinational now partly owned by GSK completed purchase of Shelys in 2014; in Kenya in 2016, Strides, an India-based multinational, took a controlling 51% interest in Universal, the only Kenyan company with WHO product-prequalification. In Uganda, Cipla, another India-based multinational, has consolidated its investment in Cipla Quality Chemicals.

Further afield, rising pharmaceutical investment in Ethiopia has included a Sino-Ethiopian joint venture between an Ethiopian distributor and China Associate Group, a company co-owned by a large group of Chinese pharmaceutical companies, and also Middle East investments (Gebre-Mariam *et al.* 2016). Local investors in Tanzania have also been actively creating start-ups. Most firms in Tanzania and Kenya remain locally owned, and when interviewed, all were strongly focused on technological upgrading to meet Good Manufacturing Practice (GMP) standards. Health sector buyers can encourage and benefit from these rising standards, while also helping to sustain domestic market competition. Manufacturers’ interviews, and contrasts between the manufacturing sector in Tanzania and Kenya, identified some of the cumulative benefits that can be generated from proximity within successful industrial clustering, including: building local skilled labour pools; generating and sharing technological knowledge and innovation skills; diversifying global linkages; achieving local specialization; improving quality of input suppliers; and generating scale economies and pressure for regional market integration (see also Schmitz 1995; Nadvi and Halder 2005).

### Aligning incentives for local health and industrial improvement

The key concerns about medicines expressed by health policy makers were quality, price and availability/reliable supply. The interviews identify areas where policies incentivizing industrial development can also incentivize responsiveness to health sector needs, and *vice versa,* extracting proximity benefits in the form of synergy between sectors.

#### Relational procurement to improve local manufacturers’ responsiveness

Health sector actors can shape manufacturing suppliers through relational procurement behaviour. The health sector provides a huge market for industry, so health sector purchasing operates implicitly as industrial policy (Chataway *et al.* 2016). The policy challenge is to generate incentives for industrial behaviour that favours health needs, reducing gaps and lead times through geographical and relational proximity while meeting quality hurdles.

Good practice exists in East Africa on working relationally with local suppliers to build responsiveness. Public and non-profit procurement agencies in Tanzania and Kenya already buy locally proportionately more essential medicines than private wholesalers (Table 2).

**Table 2.** Tanzania and Kenya 2013: Country of origin of tracer medicines, % by wholesale sector

Country of origin	Tanzania		Kenya		
	Wholesale sector		Wholesale sector		
	Public	Private	Public	FBO/NGO	Private
Tanzania	22	11			
Kenya	10	20	54	76	32
India	49	47	30	8	31
Other	18	22	16	16	37
Total	100	100	100	100	100

*Source:* Fieldwork; columns may not add to 100 because of rounding.

Not all local suppliers are responsive: in 2013, one non-profit wholesaler in Tanzania had experienced some longer local lead times than ordering from India. Experience shows these problems can be overcome by procurement that works knowledgeably and interactively with local manufacturers. The large FBO wholesaler in Kenya, Mission for Essential Drugs & Supplies (MEDS) had bought locally a high proportion of tracer medicines (Table 2), and all Kenyan faith-based health facility interviews in 2013 attributed to MEDS a high level of responsiveness, with rapid turnaround on orders. MEDS attributed this performance to relational working. Approved local suppliers were regularly inspected and monitored for delivery times and product quality, using MEDS’ pre-qualified laboratory, with sanctions for poor performance. MEDS used local tenders, and provided tender information in advance so suppliers could plan. Regular suppliers’ meetings provided feedback.

Other procurement bodies are now following this relational procurement path. The Tanzanian government has revised its procedures to permit the Tanzanian public procurement body, Medical Supplies Department (MSD), to procure directly from manufacturers, rather than solely through private distributors. In 2017, MSD described how it was building links with local firms. Close assessment of firms’ capabilities had resulted in most local firms receiving supply contracts in 2017; procurement adaptations had included smaller contract size, and 2-year contract lengths to encourage firms to invest. Firms were required to offer short delivery times, and MSD aimed to identify and share future market opportunities with local firms.

In Kenya, health sector decentralization reforms devolved public ordering of medicines and supplies to the counties, aiming to improve responsiveness to local needs. The Kenya Medical Supplies Authority (KEMSA) remains the primary public sector procurement agent, and may establish county branches (Republic of Kenya 2015: 247–248). Organization reform in KEMSA has also included framework contracts with local manufacturers, speeding up ordering under pre-negotiated terms with more active contract management (Yadav 2014).

The Global Fund procurement system,^3^ furthermore, now aims to find and work actively with potential suppliers in Africa, rewarding cost and responsiveness advantages arising from geographical proximity. The Fund engages with suppliers to identify areas for bringing in production efficiencies and reducing costs, and supports firms with market data.

Procurement agencies can also help to direct investment to priority local needs. In Tanzania, MSD is encouraging existing firms to expand their product range: in the words of one government official, fewer cough mixtures and more items of ‘real importance’. MSD is also helping to identify opportunities for new investors, and to support new start-ups with small orders. Stated national priorities included more producers of basic antibiotics such as amoxicillin, and beginning local production of laboratory reagents—in constant shortage. The local start-ups in Tanzania in 2017 included production facilities for medical supplies such as bandages, dressings and gauze, often in severely short supply, using locally produced inputs such as cotton. Currently active investments and developed proposals in the East African region also include more high-quality regional sources of ACTs and of antiretroviral medication (ARVs) for HIV; also local production of key medication for non-communicable diseases (NCDs), including hypertension and diabetes, and more regional suppliers of intravenous drips and parenteral preparations. Procurement that exploits relational proximity can thus provide a market access incentive for competent firms to respond to health sector needs.

#### Incentivizing production of both basic and higher technology products

The health sector needs affordable, good quality, secure supplies of basic items, such as basic antibiotics, pain killers and ORS, and also competent suppliers of more complex items. Incentivizing both outcomes require well-designed pricing and competition policies. Recent regional experience illustrates some of the conflicts and routes to their resolution.

East Africa-based manufacturers face sharp price competition in their domestic private markets and in bidding for tenders, since imports benefit from the EAC’s zero common external tariff for essential health supplies, while some imported inputs face duties and taxes. Africa-based manufacturers also suffer inherent cost disadvantages, notably inadequate and costly national infrastructure such as power, water and transport, forcing complementary investments, e.g. in back-up generation, and also market size constraints. Economies of scale are not large in basic formulations (tablets and capsules), but Africa-based manufacturers must import active pharmaceutical ingredients (APIs) in smaller quantities, generally at higher prices than competing Indian and Chinese firms. Local manufacturers can frequently meet competition by accepting lower margins than those earned on imports (Chaudhuri and West 2014). However, import price competition appears to have intensified, notably in basic antibiotics: Tanzanian interviewees in 2014 reported amoxicillin imports priced below API import cost. Local supplies of low margin basic essentials had dropped sharply in Tanzania, including amoxicillin (Table 3), as local firms’ business strategies refocused on higher margin products.

**Table 3.** Amoxicillin tablets/capsules of Tanzanian manufacturing origin, by sector of facility or shop (percentage of all amoxicillin found on shelves on day of visit)

Year	Public	FBO/NGO	Private	Total
2006	93	77	67	79
2009	100	81	48	74
2012	0	13	25	14
2013^a^	0	0	0	0

*Sources:* 2006; 2009; 2012: WHO/HAI primary survey data used by permission of Mary Justin-Temu.

a 2013 authors’ primary data, not a comparable sample.

Well-designed industrial protection can support prices and sustain production of essential basics, contributing to firms’ bottom line and cash flow, and also incentivize upgrading and quality improvement. It must be associated with active promotion of domestic industrial competition to prevent an upward price spiral. What is sometimes called the ‘Ghana model’ blocks imports of basic items that can be produced locally: the Ghanaian list has recently expanded to 49 medicines.^4^ It includes antibiotics, analgesics, ORS and multivitamins, and the Food and Drugs Authority (FDA) of Ghana will not accept new registrations of these medicines. East African government procurement offers local firms a percentage price uplift in public procurement: 15% in Tanzania and Uganda, varying from 10% downwards according to local ownership in Kenya, though in Kenya and Uganda manufacturing interviewees said it was not consistently applied. Other incentives that increase protection without challenging the EAC’s common tariff rules include a 2% import verification fee in Uganda, which may be raised to 12%. Tanzania has been discussing a potential list of products for local public procurement only. Kenya has a draft Trade Facilitation Act that would allow complaints by local firms alleging dumping by external suppliers.

Protecting margins on basic essentials can also provide ‘infant industry’ protection for upgrading, by giving local firms competitive breathing space to improve capabilities (Sutton 2012; West and Banda 2016). All local manufacturers interviewed were struggling to upgrade their plant, and manufacturing and quality assurance (QA) processes, to GMP standards; to expand their technical capabilities and product range; and to meet rising regulatory standards. Technical support such as that provided by German and Japanese assistance can help to exploit industrial protection to achieve rising quality. Grants, investments, and technology transfer can generate step-improvements in technological capabilities and process and product management, and reduce costs. Investments by public/private global partnerships such as Drugs for Neglected Diseases are creating one-off upgrades in locally based firms. A new start-up in Tanzania is working with German equipment suppliers to ensure high standards.

The Tanzanian government is increasingly providing land, infrastructural support and access to local longer-term loan capital, as support for new ventures. Incentivizing new investment increases domestic competition and can exert downward pressure on prices. Policy should incentivize firms’ learning to use and adapt imported technology, through effective technology transfer (Kumar *et al.* 1999) and extracting benefits from industrial clustering. Successful clusters encourage collaboration as well as competition, supporting shared technological knowledge and learning between firms (Malmberg and Maskell 2002; Ernst and Lundvall 1997).

Global Fund procurement now recognizes that to improve, learn, invest and reduce costs, local firms must sell, and it aims to reduce barriers to global market entry by competent firms in Africa. Local firms face a disincentive to apply for expensive WHO pre-qualification because they are unlikely to win tenders against Indian competition. Therefore, for prequalified firms, the Global Fund now uses a broader definition of value, called ‘total landed cost’, including points for shorter lead times and responsiveness achieved through market proximity (local firms’ most important competitive advantage). Tender success by African suppliers is increasing, including long-lasting insecticide-treated bed nets from A to Z in Arusha, a firm with in-house regional logistics, and ACTs from Cipla Quality Chemicals in Kampala. Global Fund tenders furthermore are not ‘winner takes all’: the aim is a range of competitive suppliers, and prices vary within one tender: the tender outcome sets a ‘reference price’ for an item; a single price is then paid by each country. The Global Fund’s guiding principles of value for money, quality (WHO-prequalification) and sustainability (affordability) exclude subsidies to firms; however, this procurement strategy strengthens incentives to reach international standards: ‘the carrot at the end of the [local firms’] journey’. For health systems, the journey is towards an efficient, diverse and competitive local supplier base, improving and sustainable over time.

#### Incentives to meet shared local needs: regulation and training

Policies to incentivize regulation and higher levels of pharmaceutical skills were identified as core shared health and industrial needs. Strong regulation incentivizes and supports manufacturers to reach GMP standards required for entry to donor-funded markets. Rising standards also generate merited trust in local products by clinicians and patients. Effective regulation is a shared enterprise: a complex mix of standard setting, inspection, enforcement, advice and support, checking of procured supplies, post-market vigilance and following-up users’ complaints. Manufacturers and health system actors interviewed in both research rounds agreed that external support for regulatory improvement at national and East African regional level had reduced sub-standard and counterfeit medicines in the private market, and improved quality.

The region, however, lacks key regulatory infrastructure such as high-quality reference laboratories, and needs a stronger scientific and technical base to support regulatory and training institutions. Regulatory effectiveness is uneven, with Tanzania generally recognized as having the strongest regulator, while Kenyan health sector and manufacturing interviewees were looking for regulatory improvement. Strengthening regulation incentivizes joint venture development and technology transfer: an interviewee from a multinational firm in 2017 stated that they were ‘aware of some of the key weaknesses of local pharma [in Kenya], for example around quality assurance and quality inspection procedures, and would not want to put our reputation at risk’. Regional regulatory harmonization is advocated by manufacturers to simplify intra-regional exports, and NEPAD’s Africa Medicines Regulatory Harmonisation (AMRH) provides a platform, as would the mooted Africa Medicines Agency. Local regulators can exercise closer oversight of local as compared to Indian firms, but regulatory ‘capture’ remains a danger. Supporting effective independent regulators is a key role for external actors.

Skills and training was seen by many as the area most in need of investment. Health systems need more effective supply chain and procurement management, but lack the necessary trained staff (Waako *et al.* 2009; Wiedenmayer *et al.* 2015; Yadav 2015). In 2013, 53% of Kenyan and 73% of Tanzanian health facility interviewees responsible for ordering had no relevant training. They also lack competent laboratory technicians. Nationally, medicines policy and health management need clinical pharmacists and pharmacological scientists.

These requirements overlap with the needs of industry. Pharmaceutical technicians represent one large cross-sector gap. Industrial laboratories struggle to recruit and retain skilled staff. All manufacturers cited industrial pharmacy and chemical engineering skills needs as well as biochemistry, microbiology, biomedical engineering and other allied sciences. Across the region, some tertiary institutions are introducing industry attachments, but much more is needed as the technical and scientific base for industrial growth (MIT and UNIDO 2012). Industrial development creates incentives for mutually beneficial pharmaceutical training.

Medicines policy and regulation, several stakeholders argued, must bridge the health–industry divide. A professional association official argued for the need to ‘cook our own food’ through professionalism and good regulation. Regulation of a knowledge industry such as pharmaceuticals is underpinned by science, technology and innovation; medicines policy is underpinned by clinical skills; and the two must work together locally. Skills inadequacies not only impede these ends, but, as one Kenyan respondent noted, they ‘also leave the few and over-stretched professionals available vulnerable to manipulation through corrupt practices’. The Science Technology and Innovation Strategy for Africa (STISA 2024) (African Union 2014b) speaks to this need to build regulatory and laboratory quality assurance skills through initiatives such as U.S. Pharmacopeial Convention (USP) Ghana’s Centre for Pharmaceutical Advancement and Training (CePAT). In Southern Africa, regulation and skills training are being collaboratively developed through the ZAZIBONA (Zambia, Zimbabwe, Botswana and Namibia) initiative, matching experienced with inexperienced regulators in joint inspections across the four countries, to feed into NEPAD’s AMRH programme. 

## Discussion: ‘local health’ and ‘global health’: questions of framing

Embedding local health system strengthening in local industrial development challenges silos of thought in global health. Rooted in earlier international health work that extended public health concerns across geographical boundaries (Battams and Matlin 2013), the global health literature and campaigning has generated initiatives by ‘global’—i.e., high income country-based—actors to address vast international disparities in mortality, morbidity and human wellbeing (Koplan *et al.* 2009; Rowson *et al.* 2012). These initiatives, by The Global Fund, the US President’s Emergency Plan for AIDS Relief (PEPFAR) and others, have saved huge numbers of lives and also, importantly, have reframed understanding and obligation within high-income countries.

Nevertheless, the global health field is framed and dominated by commentators, researchers, funders and campaigners based in high income countries, with associated positions of power and privilege (Horton 2014; Shiffman *et al.* 2016; Sheikh *et al.* 2017). The positionality of the global health field is reflected in its theme of globalization, of porous borders and global threats (Macfarlane *et al*. 2008). It converges with a growing literature on health security addressing cross-border fast-moving infectious diseases, HIV and biological weapons/bioterrorism, focusing public and policy attention on protecting high income populations from diseases emanating from low- and middle-income countries (Aldis 2008; Rushton 2011; Flahault *et al.* 2016). Global health actors, furthermore, have operated on the underlying assumption that medical health technologies are readily available commodities; that utilization and access can be generated in a timely manner from global pharmaceutical value chains; and that ‘global’ advances in knowledge benefit all.

For ‘local health’ actors, none of these assumptions look secure: the risk calculation is different. Diversification of supply to include competent Africa-based firms promises to reduce risk in the medium term, as do increasingly responsive local supply chains. A Kenyan interviewee argued in 2017 that emergency preparedness is a whole system challenge, including responsive suppliers and the industrial and scientific capabilities to address future challenges. Proximity and health–industrial linkages then move from irrelevance to centrality in local policy concerns.

Positionality outside high income contexts thus generates distinctive health needs and priorities, time-scales and perceptions of opportunities and risks (and risk management) in crafting robust health systems in Africa through building local capabilities. The WHO’s ‘building blocks’ for health system strengthening include access to essential medicines (WHO 2010); they do not include the industrial capabilities to supply those commodities. African policy makers are shifting however from perceiving industrial and health sector development as in competition, to perceiving symbiosis: a good example is the Tanzanian government’s recent sharp increase in budgeted tax funding for public medicines procurement, recognizing the mutual health and industrial benefits. The increasingly influential African ‘local health’ perspective outlined in this article implies a paradigm shift to embed health system strengthening within polices and narratives for inclusive and sustainable industrial development.

## Conclusion

While the African ‘local health’ perspective is distinct from global health viewpoints, it is not necessarily in contradiction, as the shifting Global Fund procurement processes illustrate. A local health framework, focused on exploiting the interrelated health and industrial benefits from proximity, throws into relief the relevance of positionality. It challenges global health actors to recognize and manage their own (large) industrial impact, and to do so in recognition of the legitimate agency of African policy makers in seeking medium term strengthening of their local health–industrial linkages and associated scientific and industrial capabilities in the interests of sustainably stronger local health systems and a stronger industrial base. From African perspectives, the huge rise in medicines procurement for SSA, arising from global health initiatives, has opened opportunities to link industrial development into strengthening their own health systems in the medium term. This article has sought to outline a ‘local health’ perspective, based in East African evidence, on some of the key opportunities to align industrial and health objectives to the cumulative benefit of both sectors.

## Supplementary Material

Supplementary Data

## Footnotes

1 Funded by DFID/ESRC, see Funding statement.

2 Funded by BMZ, see Funding statement.

3 Global Fund information from interviews, cited with permission.

4 Gazette Executive Instrument (EI) 181, 10/5/17.
