# Supplementary material for: Health-industry linkages for local health: reframing policies for African health system strengthening
Source: Health Policy Plan. 2018 Mar 19;33(4):602–10. doi: 10.1093/heapol/czy022 (PMC5894083; doi:10.1093/heapol/czy022)
Supplement: Supplementary Data [file czy022_health_industry_linkages_supplementary_material.docx]

**Health-industry linkages for local health: reframing policies for African health system strengthening**

**Supplementary materials: Lists of tracer medicines and other supplies**

**Table A1 Tracer medicines list, by country**

| **Tanzania** | **Kenya** |  |
| --- | --- | --- |
| **Medicine name and strength** | **Medicine name and strength** | **Dosage form** |
| Artemether + Lumefantrine (AL/Alu: adult); 120+20mg | Artemether + Lumefantrine (AL/Alu: adult); 120+20mg | Tablet |
|  | Artemether + Lumefantrine (AL/Alu: child); 120+20mg | Tablet |
| Sulfadoxine + Pyrimethamine; (SP) 500+25mg | Sulfadoxine + Pyrimethamine; (SP) 500+25mg | Tablet |
| Quinine; 600mg/2ml | Quinine; 600mg/2ml | Injectable |
| Amoxicillin (adult); 250mg/ 500mg | Amoxicillin (adult); 250mg/ 500mg | Tablet/capsule |
| Amoxicillin syrup (child); 125mg/5ml | Amoxicillin syrup (child); 125mg/5ml | Syrup |
| Benzyl penicillin 5000000IU (5MU) | Benzyl penicillin 5000000IU (5MU) | Injectable |
| Ciprofloxacin 250mg; 500mg | Ciprofloxacin 250mg; 500mg | Tablet/capsule |
| Atenolol; 50mg;100mg | Atenolol; 50mg; 100mg | Tablet |
| Paracetamol; 500mg | Paracetamol; 500mg | Tablet |
| Diclofenac; 50mg; 100mg | Diclofenac; 50mg; 100mg | Tablet |
|  | Morphine 50mg/ 100mg per ml | Tablet |
| Zidovidine + Lamivudine + Efivarenz  300mg+150mg+6000mg | Tenofovir + Lamivudine 300+150mg | Tablet |
| Zidovidine + Lamivudine + Nevirapine; 399mg+150mg+200mg | Nevirapine; 200mg | Tablet |
| Tenofovir + Entricitabine + Lopinavir  200mg+200mg+250mg | Lopinavir/ Ritonavir 200mg/50mg | Tablet |
|  | Zidovudine; 300mg | Tablet |
| Oxytocin; 10 iu & 5iu per m | Oxytocin; 10 iu & 5iu per m | Injectable |
| Metronidazole; 200mg; 400mg | Metronidazole; 200mg; 400mg | Tablet |
| Fluconazole; 50mg; 150mg; 200mg | Fluconazole; 50mg; 150mg; 200mg | Tablet |
|  | Albendazole; 200mg/ 400mg | Tablet |
| Mebendazole; 100mg | Mebendazole; 100mg | Tablet |
| Omeprazole 20mg | Omeprazole 20mg; 40mg | Tablet |
| Clotrimazole cream; 1% | Clotrimazole cream; 1% | Cream |
|  | Ketoconazole 50mg | Tablet |
| Amitriptylline; 25mg | Amitriptylline; 25mg | Tablet |
| Metformin 500mg | Metformin 500mg; 850mg | Tablet |
| Glibenclamide 5mg | Chlorpromazine;25mg; 100mg | Tablet |
| Loperamide hydrochloride 2mg | Loperamide hydrochloride 2mg | Tablet |
| Normal saline and 5% Dextrose (IV fluid) | Normal saline and 5% Dextrose (IV fluid) | Infusion |

**Table A2 Other supplies tracer list, by country**

| **Tanzania** | **Kenya** |
| --- | --- |
| **Medical equipment** |  |
| Thermometer | Thermometer |
| Blood Pressure Machine | Blood Pressure Machine |
| Microscope | Microscope |
| Stethoscope | Stethoscope |
| Foetoscope for midwifery | Foetoscope for midwifery |
| Glucometer | Glucometer |
| Weighing scales for paediatrics | Weighing scales for paediatrics |
| CD4 machine | CD4 machine |
| **Medical supplies** |  |
| Slides for the microscope | Slides for the microscope |
| Strips for the glucometer | Strips for the glucometer |
| Sharps box | Sharps box |
| Surgical gloves | Clinical gloves |
| Gauze bandages | Gauze bandages |
| Crepe bandages | Crepe bandages |
| Syringes and needles | Syringes and needles |
| Hydrogen peroxide (H2O2) | Hydrogen peroxide (H2O2) |
| Alcohol/Spirit for wound cleaning | Alcohol/Spirit for wound cleaning |
| **Laboratory supplies** |  |
| Haemoque for HB level | Urine test strips |
| Gram stain reagent for bacterial infection | Pregnancy test strips |
| Giemsa stain | Giemsa stain |
| Emulsion oil | Emulsion oil |
| Determine HIV test kit | Determine HIV test kit |
|  | Unigold HIV test kit |
| SD Bioline test for syphilis | Bioline HIV test kit |
| Rapid diagnostic test for malaria | Rapid diagnostic test for malaria |
|  | Widal reagent |
|  | EDTA tubes |
| **Other essential supplies** |  |
| Disinfectants (Hibitane or Savlon) | Disinfectants (Hibitane or Savlon) |
| Mackintoshes/ plasticised sheeting | Mackintoshes/ plasticised sheeting |
| Bed net | Bed net |
| Bed sheets | Bed sheets |
| Mop or broom | Mop or broom |
| Detergents |  |
